# Corrigendum to “Recommendations for Diagnosis and Management of Osteoporosis in COPD Men”

**DOI:** 10.1155/2017/2532464

**Published:** 2017-07-02

**Authors:** Elias E. Mazokopakis, Ioannis K. Starakis

**Affiliations:** ^1^Department of Internal Medicine, Naval Hospital of Crete, Chania, 73 200 Crete, Greece; ^2^Department of Internal Medicine, Patras University Hospital, 26500 Rion-Patras, Greece

In the article titled “Recommendations for Diagnosis and Management of Osteoporosis in COPD Men” [1], there was an error regarding the FRAX® tool, which should be clarified as follows.

The article notes: “For example, the Garvan fracture risk calculator and the WHO fracture risk assessment tool (FRAX) are useful supplements to BMD assessments, as they help physicians to decide which patients might require prolonged treatment to reduce the risk of future fractures [24, 25]”; “The updated National Osteoporosis Foundation (NOF) guide based upon the WHO fracture prediction algorithm (FRAX), in conjunction with an updated US-specific economic analysis, provides also treatment recommendations for men with osteopenia on BMD.” However, the World Health Organization (WHO) did not develop, test, or endorse the FRAX tool or its recommendations [2]. The metabolic bone disease unit at the University of Sheffield that developed FRAX was a WHO Collaborating Centre from 1991 to 2010, but treatment guidelines must undergo a formal process before they can be endorsed by the WHO.

## References

[1] Mazokopakis E. E., Starakis I. K. (2011). Recommendations for diagnosis and management of osteoporosis in COPD men. *ISRN Rheumatology*.

[2] Ford N., Norris S. L., Hill S. R. (2016). Clarifying WHO’s position on the FRAX® tool for fracture prediction. *Bulletin of the World Health Organization*.

